# Anti-SARS-CoV-2 Revaccination Success in Kidney Transplant Recipients With No Initial Humoral Response Is Linked to Primary Vaccine Type

**DOI:** 10.3389/fmed.2022.910987

**Published:** 2022-07-04

**Authors:** Julian Stumpf, Jörg Schwöbel, Claudia Karger, Holger Schirutschke, René Mauer, Anna Klimova, Torsten Tonn, Christian Hugo

**Affiliations:** ^1^Medizinische Klinik und Poliklinik III, Universitätsklinikum Carl Gustav Carus, Technische Universität Dresden, Dresden, Germany; ^2^Kuratorium für Heimdialyse (KfH)-Nierenzentrum Dresden, Dresden, Germany; ^3^Dialysezentrum Chemnitz, Chemnitz, Germany; ^4^Kuratorium für Heimdialyse (KfH)-Nierenzentrum am Klinikum St. Georg, Leipzig, Germany; ^5^Patienten-Heimversorgung Gemeinnützige Stiftung (PHV) Dialysezentrum Dresden Friedrichstadt, Dresden, Germany; ^6^Faculty of Medicine Carl Gustav Carus, Institute for Medical Informatics and Biometry, Technische Universität Dresden, Dresden, Germany; ^7^National Center for Tumor Diseases Dresden, Dresden, Germany; ^8^Institute for Transfusion Medicine, German Red Cross Blood Donation Service North-East, Dresden, Germany; ^9^Faculty of Medicine Carl Gustav Carus, Transfusion Medicine, Technische Universität Dresden, Dresden, Germany

**Keywords:** revaccination, kidney transplant recipient (KTR), SARS-CoV-2, humoral response, 1273-mRNA, BNT162b2-mRNA, clinical decision making, guidelines

## Abstract

**Background:**

While anti-SARS-CoV-2 vaccination success in kidney transplant recipients (KTR) after two doses and 1273-mRNA was associated with higher seroconversion rates compared to BNT162b2-mRNA in our “DIA-Vacc Study” (NCT04799808), it remains unclear whether this may also be the case in non-responding KTR after a third vaccination dose.

**Materials and Methods:**

Non-responding KTR (after two mRNA vaccinations) were investigated 4.5–6 months after study enrollment at first vaccination. One hundred sixty-six of 193 received a third vaccination between 3.5 and 5 months after the initial study enrollment and were always investigated 4 weeks later, exploring humoral immune response (ELISA) and specific cellular responses (interferon-γ release assay). Sixty-seven of 193 measurements in KTR were done immediately before the third vaccination or in KTR without further vaccination at 4.5–6 months.

**Results:**

Of 193 KTR with no initial immune response 4 weeks after the second vaccination, 106/87 were immunized twice with 1273-mRNA/BNT162b2-mRNA, respectively. Additional mRNA booster vaccination led to positive seroconversion rates of 30–50%, while 16% of the initial non-responders demonstrated a delayed seroconversion without any booster vaccination. Using logistic regression analysis, a positive IgG response after the third vaccination was 23% more likely if the primary vaccine type was 1273-mRNA compared to BNT162b2-mRNA (OR = 4.420, 95% CI [1.208–16.173], *p* = 0.025). Primary vaccine type, a weak anti-SpikeS1 IgG response 4 weeks after second vaccination (3.2–35.2 BAU/ml, *p* < 0.001) and a lack of MMF/MPA as part of the immunosuppressive treatment (trend, *p* = 0.06) but no other variables studied correlated with seroconversion success.

**Conclusion:**

This observational study adds important evidence toward using 1273-mRNA as the primary mRNA vaccine type for immunosuppressed KTR.

## Introduction

SARS-CoV-2 (Severe Acute Respiratory Syndrome Corona Virus-2) causes COVID-19 disease. More than 2 years have passed since its initial discovery in Wuhan, China in December 2019, and SARS-CoV-2 infection has rapidly evolved into an international pandemic with devastating consequences (1, 2). Seroconversion rates for the general population after two doses of mRNA vaccination (3, 4) and the usefulness of a third vaccine booster dose, particularly for protection against new viral variants (5) have been reported. Others and we demonstrated that kidney transplant recipients (KTR) have a markedly decreased seroconversion rate after two doses of mRNA vaccination (6, 7) resulting in reduced protection against COVID-19. On the other hand, due to higher mortality in KTR, successful vaccination to protect against COVID-19 disease is crucial for this population. It is worth noting that vector vaccines (such as CoronaVac) have even lower seroconversion rates (8). Short-term seroconversion rates in 2x mRNA vaccinated but non-responding KTR receiving the third vaccination with mRNA vaccine varies between a third (9) and a half (10, 11) but side-by-side comparisons of 1273-mRNA and BNT162b2-mRNA are lacking. While 1273-mRNA was associated with higher seroconversion rates after two vaccinations compared to BNT162b2-mRNA in our observational, multicenter cohort DIA-Vacc study (6), it remains unclear whether this may also be the case in non-responding KTR after a third vaccination. We also asked the question, of whether vaccine type for the initial two vaccinations or the third “booster” vaccination is more relevant for seroconversion success. Within the DIAVacc study cohort, we now report seroconversion rates after an approximately half-year in non-responsive KTR who either did not receive another booster vaccination or were exposed to a third vaccination using either 1273-mRNA, BNT162b2-mRNA, or vector vaccines in various combinations.

## Materials and Methods

### Contextual Information

Background information of the underlying DIA-Vacc study (NCT number: 04799808), which investigated the time point 8 weeks after the first vaccination, has already been published elsewhere (6). However, as some parts of it are indispensable for the understanding and interpretation of the present work, excerpts from it are included here together with new notes. In all DIA-Vacc study vaccination participants (eligibility if >18 years old and signed informed consent) SARS-CoV-2 antibody formation was analyzed. Previous or current COVID-19 disease, specific IgG- or IgA-antibodies against the Spike protein S1 (*de novo* development as the primary study aim) and IgG-antibodies against the nucleocapsid protein subunit (NCP, to exclude previous and current infection), as well as antibodies against the receptor-binding domain (RBD), were assessed. In a representative subcohort, interferon-γ release assays (IGRA) were done to analyze the development of a T-cellular immune response after vaccination/disease. The study time points were before (T0), 8 weeks (T2), and 6 months (T3) after the start of vaccination (6). In the observational DIA-Vacc study, medical personnel, dialysis patients, and KTR were vaccinated against SARS-CoV-2 using either BNT162b2- or 1273-mRNA. The first vaccination dose was administered between 15 January and 24 February, followed by a second dose 3 or 4 weeks later, depending on the vaccine type. Only the first 26 of 36 nephrology centers, providing 3,101 participants, were accepted for the study due to funding restrictions. By vaccine availability during January (BNT162b2-mRNA) and February (1273-mRNA) 2021, only the first four dialysis centers assigned to the vaccination campaign, received BNT162b2-mRNA, while all the other following dialysis centers received 1273-mRNA vaccine for both (first and second dose) vaccinations. Neither any dialysis center nor any participant nor the study center (Dresden) had a choice or influence regarding the type of vaccine, which was assigned in the order of contacting the central vaccination institute in Saxony. The central vaccination institute distributed information about the start of the vaccination campaign *via* email at the same time to all dialysis centers.

### Current Information

**FIGURE 1:**
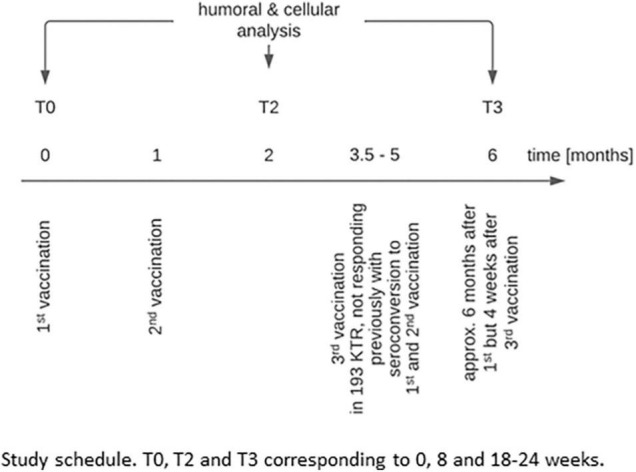
Study schedule. T0, T2, and T3 correspond to 0, 8, and 18–24 weeks.

In the study presented here, we analyzed KTR who did not show a *de novo* positive humoral response at T2 (10) as defined by either IgG- or IgA- anti-SpikeS1 antibodies to the first and second mRNA vaccination. An optional third vaccination was offered between 3.5 and 5 months after T0 and always investigated 4 weeks later, targeting the highest humoral response (Figure 1). Since at that time no recommendation for a third vaccination was given by the German national authorities, the decision for an additional booster vaccination and choice of vaccine-type was in the hands of the dialysis centers. In addition to the mRNA vaccines BNT162b2-mRNA and 1273-mRNA, a vector vaccine was used in eight cases as a third dose after two vaccinations with mRNA vaccines (5x AZD1222 and 3x Ad26.COV2.S). COVID-19 diseased patients (symptomatically and asymptomatically, the latter being assessed by NCP seroconversion), during and after vaccination (up to T3) were excluded to evaluate the vaccination-related immune response. Patients were tested for SARS-CoV-2 infection by RT-PCR in the dialysis centers, if they presented one of the classic symptoms (fever, cough, shortness of breath, myalgias, diarrhea, or other symptoms consistent with such an infection) or if they were in contact with a person with RT-PCR-confirmed disease. Routine PCR screening without a cause was not part of the good medical practice of the dialysis centers.

For all antibody measurements, Euroimmun ELISAs on Euroimmun analyzers were used (12–16).

### Endpoints

**FIGURE 2:**
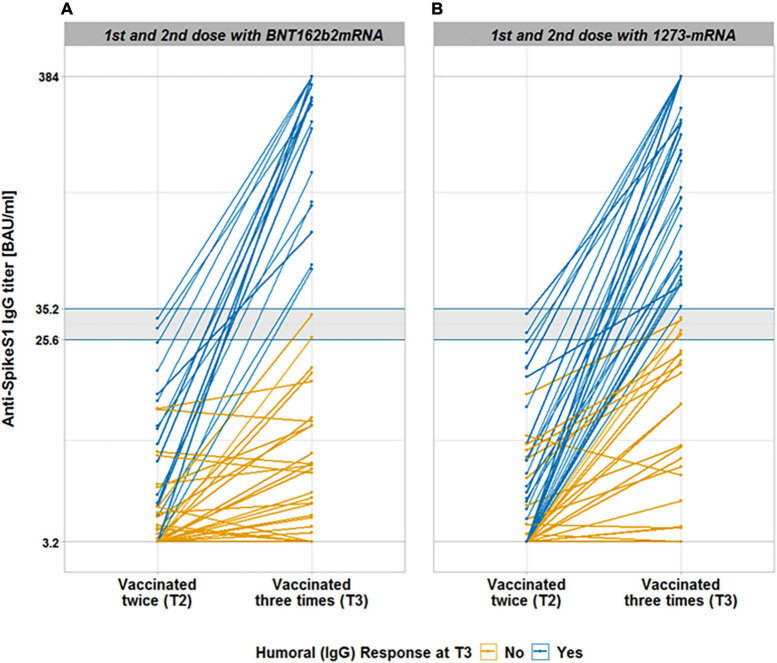
Anti-SpikeS1 IgG antibody titer after 3rd vaccination in 193 kidney transplant recipients, not responding previously with seroconversion to 1st and 2nd vaccination. This figure shows anti-SpikeS1 IgG antibody titers after the 2nd and 3rd vaccination, depending on the vaccine type of the 1st and 2nd vaccine type being BNT162b2-mRNA **(A)** and 1273-mRNA **(B)**. The gray lines indicate the upper and lower threshold of the test (384 and 3.2 BAU/ml), respectively. Colored horizontal lines enclose and delineate the gray area for anti-SpikeS1 IgG antibody titer positivity (25.6 and <35.2 BAU/ml). The gray area still indicates a lack of seroconversion. A “weak” reaction (>3.2 and <35.2 BAU/ml) was distinguished from a negative reaction, the latter indicating 3.2 BAU/ml, in our statistical approach, while all values below 25.6 BAU/ml represent a negative antibody reaction according to the manufacture leaf. Blue progress lines indicate the antibody titer of kidney transplant recipients who seroconverted after a 3rd dose, whereas yellow progress lines indicate a lack of seroconversion.

The primary endpoint of the study was the positive humoral immune response 4 weeks after a third vaccination dose as defined by *de novo* positivity of either IgG- or IgA- anti-SpikeS1 antibodies (Table 1 and Figure 2) without the development of virus-specific NCP antibodies. Secondary endpoints were the development of vaccination-induced serological or T-cellular response parameters and titers.

**TABLE 1:** Humoral and T-cellular response rates 6 months (T3) after the 1st vaccination.

Variable	Category	3x BNT162b2	2x BNT162b2, 1x 1273	2x 1273, 1x BNT162b2	3x 1273	2x mRNA, 1x vector	*p*_Value	2x mRNA
Patient number	N	57	22	16	63	8		67
**Humoral response**								
IgG-Ab or IgA-Ab SpikeS1 positive	n of total n (%)	17/57 (29.8%)	8/22 (36.4%)	8/16 (50%)	31/63 (49.2%)	1/8 (12.5%)	0.092	11/67 (16.4%)
IgG-Ab Spike S1 positive	n of total n (%)	12/57 (21.1%)	6/22 (27.3%)	7/16 (43.8%)	29/63 (46%)	1/8 (12.5%)	0.025	10/67 (14.9%)
RBD positive	n of total n (%)	11/57 (19.3%)	5/22 (22.7%)	3/16 (18.8%)	20/63 (31.7%)	1/8 (12.5%)	0.464	6/67 (9%)
IgA-Ab SpikeS1 positive	n of total n (%)	12/57 (21.1%)	6/22 (27.3%)	4/16 (25%)	21/63 (33.3%)	1/8 (12.5%)	0.524	7/67 (10.4%)
**Interferon-γ release assay (IGRA)—T-cellular response**								
IGRA positive	n of total n (%)	1/16 (6.2%)	0/7 (0%)	0/0 (0%)	6/16 (37.5%)	1/7 (14.3%)	0.06	5/25 (20%)

Humoral vaccination responses were assessed as positive when de novo production of the anti-SpikeS1 IgG and (anti-SpikeS1 IgA or IgG endpoint of the original DIA-Vacc study)/or IgA or IgG protein or anti-RBD IgG subunit was above positivity level. A positive T-cellular response to vaccination as assessed by interferon-γ release assay (IGRA) turned from a negative result on T0 to positive on T3, respectively (≥100 mIU/ml, as being recommended by the manufacturers). For this evaluation, all participants with asymptomatic* or documented symptomatic** COVID-19 disease before and during vaccination up to T3 (6 months) were excluded.

*BNT162b2 = BNT162b2-mRNA or tozinameran or brand name Comirnaty; 1273 = 1273-mRNA or brand name Spikevax.*

*Asymptomatic COVID-19 disease definition–neither knowledge nor symptoms of COVID-19 disease, but IgG-antibody reaction to nucleocapsid (T0, T2, or T3) or the Spike protein subunit S1 (only T0) of the SARS-CoV-2 virus is positive.

**Symptomatic COVID-19 disease definition–SARS-CoV-2 PCR positive patients with clinical symptoms.

**FIGURE 3:**
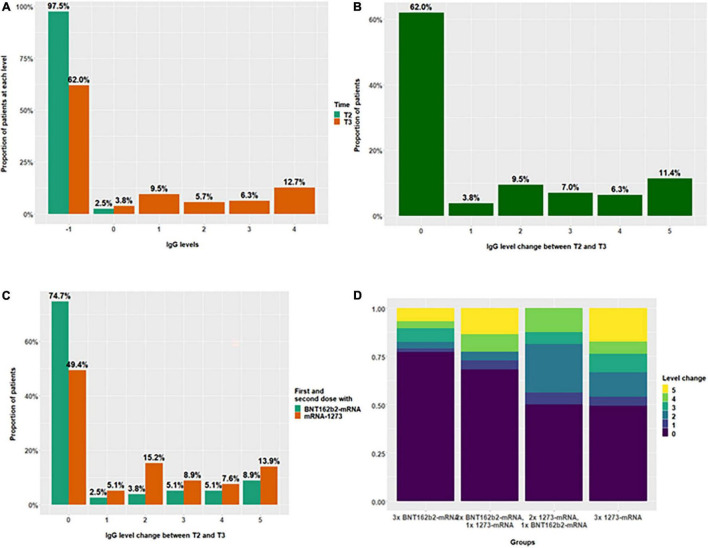
Distribution of anti-SpikeS1 IgG level change after the 3rd vaccination in 193 kidney transplant recipients, not responding previously with seroconversion to the 1st and 2nd vaccination. The distributions of IgG levels according to interval categorizations at T2 and T3 **(A)**, as well as their change after the third vaccination are summarized in panels **(B–D)**. Level “–1” is assigned to negative test values (anti-SpikeS1 IgG < 25.6 BAU/ml). Values below the corresponding positivity threshold (35.2 BAU/ml) but above the threshold for negativity were assigned to level “0”. The remaining test values were divided into four intervals of approximately equal length (level “1” <100 and ≥35.2; level “2” <200 and ≥100; level “3” <300 and ≥200; level “4” ≥300). These intervals can be used to quantify the change of IgG levels between T2 and T3, where, for example, a positive change corresponds to an increase in IgG, with a change of five being the maximum increase, which occurred in 11.4% **(B)**. Level changes (proportions of patients) between T2 and T3 depending on the vaccine used for 1st and 2nd vaccination are shown in panel **(C)**, whereas, the proportions with regard to the different vaccine combinations are shown in panel **(D)**.

**FIGURE 4:**
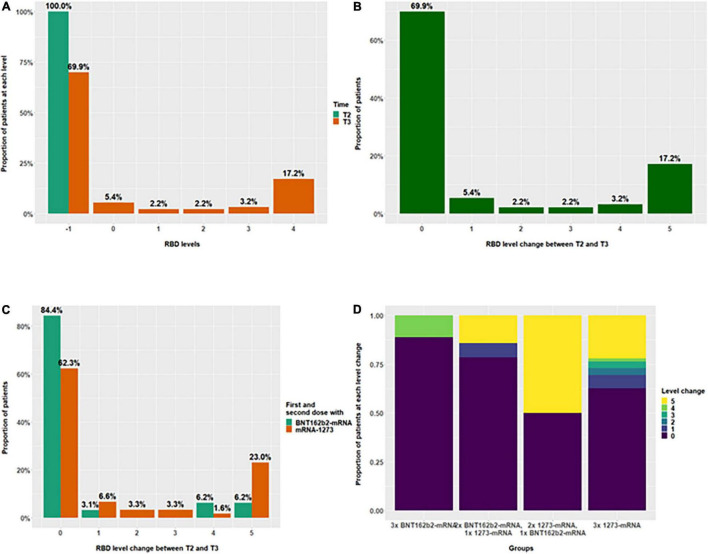
Distribution of anti-RBD IgG level change after the 3rd vaccination in 193 kidney transplant recipients, not responding previously with seroconversion to the 1st and 2nd vaccination. The distributions of RBD levels according to interval categorizations at T2 and T3 **(A)**, as well as their change after the third vaccination are summarized in Figures 5B–D. Level “–1” is assigned to negative test values (anti-RBD IgG < 20% inhibition [IH]). Values below the corresponding positivity threshold (35% IH) but above the threshold for negativity were assigned to level “0”. The remaining test values were divided into four intervals of approximately equal length (level “1” <50 and ≥35; level “2” <65 and ≥50; level “3” <80 and ≥65; level “4” ≥80). These intervals can be used to quantify the change of RBD levels between T2 and T3, where, for example, a positive change corresponds to an increase in RBD, with a change of five being the maximum increase, which occurred in 17.2% **(B)**. Level changes (proportions of patients) between T2 and T3 depending on the vaccine used for 1st and 2nd vaccination are shown in panel **(C)**, whereas, the proportions with regard to the different vaccine combinations are shown in panel **(D)**.

Trends of antibody and IGRA titers (Table 2) are described in more detail at the end of the corresponding Table 3, Figure 3A (anti-SpikeS1 IgG), and Figure 4A (anti-RBD IgG). Likewise explained is the interval categorization (referred to as “levels” in the data analysis) to analyze the effect of the vaccines on the exact anti-SpikeS1 and –RBD IgG titer levels. In addition, change in levels between T2 and T3, varying from 0 to 5, was calculated for each patient (Figure 3B for anti-SpikeS1 and Figure 4B for anti-RBD IgG) and the dependence on the type of vaccine (Figure 3C for anti-SpikeS1 and Figure 4C for anti-RBD IgG) and the different vaccine combinations (Figure 3D for anti-SpikeS1 and Figure 4D for anti-RBD IgG) was investigated.

**TABLE 2:** Humoral and T-cellular titers at study time points two (T2) and six (T3) months in different vaccine combinations.

Var	Type of vaccines	Category	T2	T3	*P*-value
IgG-Ab Spike S1	3x BNT162b2	Median (IQR)	3.2 (3.2–4.6)	3.8 (3.2–16.7)	<0.001
	2x BNT162b2, 1x 1273	Median (IQR)	3.2 (3.2–3.2)	3.5 (3.2–52.5)	0.003
	2x 1273, 1x BNT162b2	Median (IQR)	3.2 (3.2–6.2)	22.8 (3.2–52.6)	0.003
	3x 1273	Median (IQR)	3.2 (3.2–5)	28.1 (3.2–209.4)	<0.001
	2x mRNA, 1x vector vaccine	Median (IQR)	3.2 (3.2–3.2)	3.2 (3.2–6)	0.181
	2x mRNA	Median (IQR)	3.2 (3.2–6.4)	3.6 (3.2–21.9)	
RBD-IgG-Ab RBD	3x BNT162b2	Median (IQR)	3.8 (3.2–6.2)	10.2 (7.6–15.3)	0.088
	2x BNT162b2, 1x 1273	Median (IQR)	1.4 (1.1–4.8)	3.6 (1.6–21)	0.093
	2x 1273, 1x BNT162b2	Median (IQR)	0 (0–5.3)	4.2 (0–29.9)	0.5
	3x 1273	Median (IQR)	3.9 (1.9–7.7)	7.5 (0–58.2)	<0.001
	2x mRNA, 1x vector vaccine	Median (IQR)	2.7 (0–3.4)	0 (0–1.1)	0.529
	2x mRNA	Median (IQR)	4.5 (1.9–7.7)	3.9 (0–9.4)	
IgA-Ab Spike S1	3x BNT162b2	Median (IQR)	0.3 (0.2–0.5)	0.4 (0.2–0.7)	0.156
	2x BNT162b2, 1x 1273	Median (IQR)	0.4 (0.2–0.7)	0.5 (0.3–1.3)	0.001
	2x 1273, 1x BNT162b2	Median (IQR)	0.3 (0.2–0.3)	0.3 (0.2–0.7)	0.289
	3x 1273	Median (IQR)	0.3 (0.2–0.5)	0.5 (0.3–1.4)	<0.001
	2x mRNA, 1x vector vaccine	Median (IQR)	0.3 (0.1–0.4)	0.3 (0.2–0.4)	0.313
	2x mRNA	Median (IQR)	0.4 (0.2–0.6)	0.3 (0.2–0.6)	
Interferon-γ release assays (IGRA)	3x BNT162b2	Median (IQR)	13.3 (0.9–38.9)	8.3 (1.3–60.3)	0.266
	2x BNT162b2, 1x 1273	Median (IQR)	0 (0–0.1)	17.2 (5.5–43.4)	0.5
	2x 1273, 1x BNT162b2	Median (IQR)	29.3 (29.3–152.8)		
	3x 1273	Median (IQR)	23.6 (3.7–151.4)	28.6 (6.1–200.4)	0.952
	2x mRNA,1x vector vaccine	Median (IQR)	7.1 (0–183.6)	11.9 (7.1–31.3)	0.062
	2x mRNA	Median (IQR)	26.1 (11.8–444.3)	23.7 (5.8–107)	

This table compares the average titer levels (median/interquartile range = IQR) on T3 with T2 (different columns) for the different anti-SpikeS1 IgA, IgG, RBD-IgG antibodies as well as for cellular immunity via Interferon-γ release assay = IGRA measurements in patients who received different combinations of mRNA vaccine (different rows). For this evaluation, all participants with asymptomatic* or documented symptomatic** COVID-19 disease before and during vaccination up to T3 (6 months) were excluded.

*BNT162b2 = BNT162b2-mRNA or tozinameran or brand name Comirnaty; 1273 = 1273-mRNA or brand name Spikevax.*

*Asymptomatic COVID-19 disease definition–neither knowledge nor symptoms of COVID-19 disease, but IgG-antibody reaction to nucleocapsid (T0, T2, or T3) or the Spike protein subunit S1 (only T0) of the SARS-CoV-2 virus is positive.

**Symptomatic COVID-19 disease definition–SARS-CoV-2 PCR positive patients with clinical symptoms.

**TABLE 3:** Interval categorization into “level” of anti-SpikeS1-IgG and anti-RBD ranges of all participants.

Level	Interval [unit]	Participants at T2	Participants at T3

IgG level	Interval [BAU/ml]	*N*	*N*
–1	IgG < 25.6	154	98
0	25.6 ≤ IgG < 35.2	4	6
1	35.2 ≤ IgG < 100	0	15
2	100 ≤ IgG < 200	0	9
3	200 ≤ IgG < 300	0	10
4	IgG ≥ 300	0	20

**RBD level**	**Interval [% inhibition]**	***N***	***N***

–1	RBD < 20	93	65
0	20 ≤ RBD < 35	0	5
1	35 ≤ RBD < 50	0	2
2	50 ≤ RBD < 65	0	2
3	65 ≤ RBD < 80	0	3
4	RBD ≥ 80	0	16

*The detectable ranges of anti-SpikeS1 and –RBD IgG antibody values are categorized into six intervals, labeled from –1 to 4 (referred to as “levels” in the data analysis). The limit of the “–1” level is defined by the manufacturer’s test limit on negativity. The limit of the next higher level “0” follows directly upwards and includes the gray area of the corresponding test (below the positivity threshold). The limits of the other levels are chosen arbitrarily (“1,” “2,” “3,” and “4”) and represent the remaining linear test range in approximately equal intervals.*

### Statistical Analysis

In the descriptive analysis of the main study endpoints, categorical variables were summarized as absolute frequencies or percentages, and continuous variables were summarized using the mean and standard deviation or median and interquartile range (IQR). Time trends in anti-SpikeS1 IgG and –RBD IgG responses, as well as between-group differences, were analyzed either by the Wilcoxon signed-rank test, Mann–Whitney U test, or the chi-squared test, as appropriate.

**FIGURE 5:**
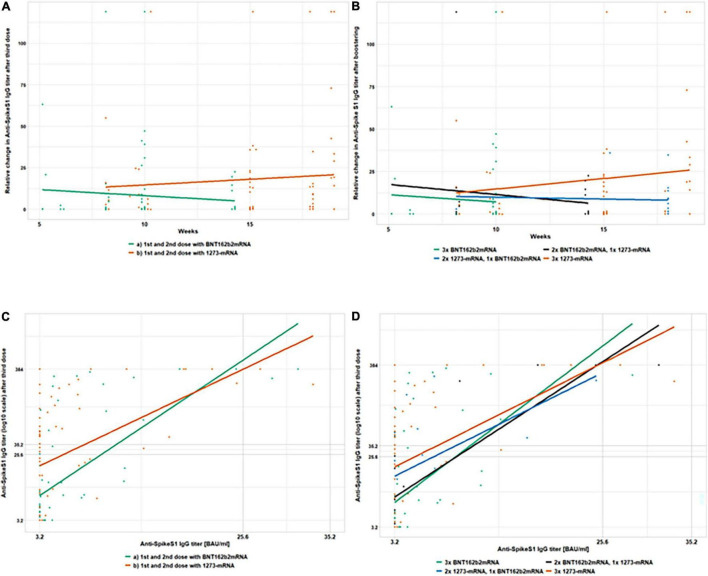
Absolute and relative changes in anti-SpikeS1 IgG antibody titer after the 3rd vaccination in 193 kidney transplant recipients, not responding previously with seroconversion to the 1st and 2nd vaccination. This figure shows absolute and relative changes in anti-SpikeS1 IgG antibody titers between the 2nd and 3rd vaccination. At T2, 4 weeks after the second vaccination dose and T3, 3.5–5 months after the first but always 4 weeks after the third vaccination dose in 193 kidney transplant recipients, anti-SpikeS1 IgG titers are depicted in four different sub-panels. The relative change in antibody titers after a third dose vs. the number of weeks passed between the second and third dose, as well as its dependence on primary **(A)** and booster **(B)** vaccine types, are shown. In panel **(A)** the horizontal progression of the orange (1273-mRNA) and green (BNT162b2) lines indicate the lack of dependence on the timing of booster vaccination. The height indicates the titer levels 4 weeks after the third vaccination. The same applies to the horizontal courses of the green (3x BNTb2 mRNA), black (2x BNT162b2 mRNA, 1x 1273 mRNA), blue (2x 1273 mRNA, 1x BNT162b2 mRNA), and orange (3x 1273 mRNA) lines in panel **(B)** which also show a lack of dependence on the time of booster vaccination. Again, the heights represent the titer heights 4 weeks after the third vaccination. In panels **(C,D)** anti-SpikeS1 IgG titers after the second dose of vaccinations (despite being below positivity level) were plotted against titers after the third dose of vaccination to examine titer dependencies as predictors of an immune response. Gray lines enclose the upper and lower threshold of the test (384 and 3.2 BAU/ml, respectively), as well as the gray area of the test (25.6 and 35.2 BAU/ml). In panel **(C)** the incline of the orange (1273-mRNA) and green (BNT162b2) lines indicate a booster success correlation to a weak compared to no anti-SpikeS1 IgG reaction after two vaccination. The height indicates the titer levels and thus the strength of seroconversion 4 weeks after the third vaccination. The same applies to the incline of the green (3x BNTb2 mRNA), black (2x BNT162b2 mRNA, 1x 1273 mRNA), blue (2x 1273 mRNA, 1x BNT162b2 mRNA), and orange (3x 1273 mRNA) lines in panel **(D)** which also show a booster success correlation to a weak compared to no anti-SpikeS1 IgG reaction after two vaccination. Heights again indicate the titer levels and thus the strength of seroconversion 4 weeks after the third vaccination.

**FIGURE 6:**
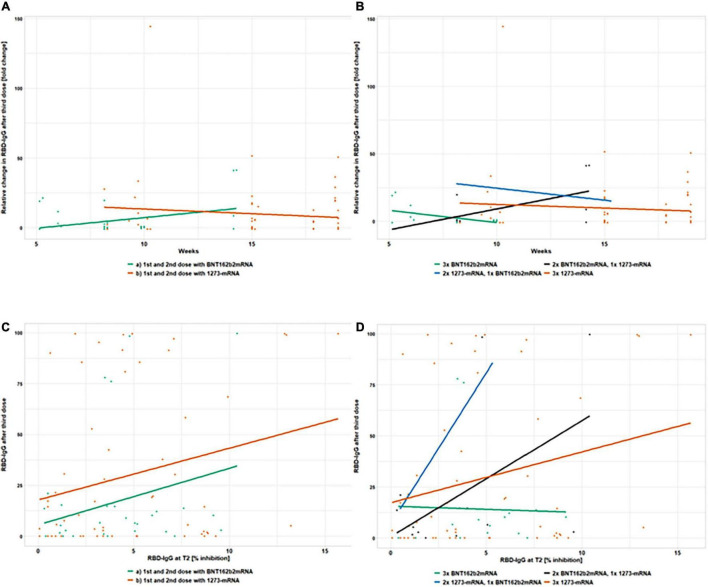
Absolute and relative changes in anti-RBD IgG antibody titer after the 3rd vaccination in 193 kidney transplant recipients, not responding previously with seroconversion to the 1st and 2nd vaccination. This figure shows absolute and relative changes in anti-RBD IgG antibody titers between the 2nd and 3rd vaccination. At T2, 4 weeks after the second vaccination dose and T3, 3.5–5 months after the first but always 4 weeks after the third vaccination dose in 193 kidney transplant recipients, anti-RBD IgG titers are depicted in four different sub-panels. The relative change in antibody titers after a third dose vs. the number of weeks passed between the second and third dose, as well as its dependence on primary **(A)** and booster **(B)** vaccine types, are shown. In panel **(A)** the horizontal progression of the orange (1273-mRNA) and green (BNT162b2) lines indicate the lack of dependence on the timing of booster vaccination. The height indicates the titer levels 4 weeks after the third vaccination. The same applies to the horizontal courses of the green (3x BNTb2 mRNA), black (2x BNT162b2 mRNA, 1x 1273 mRNA), blue (2x 1273 mRNA, 1x BNT162b2 mRNA), and orange (3x 1273 mRNA) lines in panel **(B)** which also show a lack of dependence on the time of booster vaccination. Again, the heights represent the titer heights 4 weeks after the third vaccination. In panels **(C,D)** anti-RBD IgG titers after the second dose of vaccinations (despite being below positivity level) were plotted against titers after the third dose of vaccination to examine titer dependencies as predictors of an immune response. Gray lines enclose the upper and lower threshold of the test (100 and 0% inhibition, respectively). In panel **(C)** the incline of the orange (1273-mRNA) and green (BNT162b2) lines indicate a booster success correlation to a weak compared to no anti-RBD IgG reaction after two vaccination. The height indicates the titer levels and thus the strength of seroconversion 4 weeks after the third vaccination. The same applies to the incline of the green (3x BNTb2 mRNA), black (2x BNT162b2 mRNA, 1x 1273 mRNA), blue (2x 1273 mRNA, 1x BNT162b2 mRNA), and orange (3x 1273 mRNA) lines in panel **(D)** which also show a booster success correlation to a weak compared to no anti-RBD IgG reaction after two vaccination. Heights again indicate the titer levels and thus the strength of seroconversion 4 weeks after the third vaccination.

As was observed in a number of studies (17, 18), a substantial difference in seroconversion response may occur after administering different vaccines. The analysis of IgG seroconversion predictors was carried out using multiple logistic regression analysis (Table 4). Hereby, we included the vaccine type from the first/second as well as the third vaccination as potential predictors. The relative change in antibody titers after a third dose vs. the number of weeks passed between the second and third dose, as well as its dependence on primary and booster vaccine types, are shown in Figures 5A,B (anti-SpikeS1) and Figures 6A,B (anti-RBD IgG). Anti-SpikeS1 IgG (Figures 5C,D) and anti-RBD IgG (Figures 6C,D) titers after the second dose of vaccinations (despite being below positivity level) were plotted against titers after the third dose of vaccination to examine titer dependencies as predictors of an immune response. As other aspects could also influence seroconversion rates, we also investigated gender, age, time after transplantation, and hepatitis B vaccination failure on dialysis, as well as immunosuppressive therapy with MMF/MPA and the comorbidity diabetes mellitus.

**TABLE 4:** Achieving humoral IgG response after booster vaccination (logistic regression).

Risk factor	OR	95% CI	*P-value*
Anti-SpikeS1 IgG after two doses	1.360	[1.167,1.584]	<0.001
Time between 2nd and 3rd doses	0.972	[0.855,1.106]	0.668
1st and 2nd dose with 1273-mRNA (ref = BNT162b2-mRNA)	4.420	[1.208,16.173]	0.025
3rd dose with 1273-mRNA (ref = BNT162b2-mRNA)	1.080	[0.537,2.171]	0.830
Sex (ref = female)	1.339	[0.547,3.278]	0.523
Age	0.975	[0.946,1.005]	0.107
Time after transplantation (years)	1.028	[0.954,1.106]	0.471
HepB vaccination failure	1.502	[0.231,9.748]	0.670
MMF/MPA (ref = yes)	7.086	[0.917,54.730]	0.060
Diabetes mellitus (ref = yes)	0.610	[0.182,2.044]	0.423

*Logistic regression on achieving anti-SpikeS1 IgG response 4 weeks after 3rd SARS-CoV-2 vaccination. MMF-MPA, mycophenolate mofetil or mycophenolic acid.*

For hypothesis testing, a significance level of 5% (two-sided) was chosen. Data analysis was implemented in the R Environment for Statistical Computing (19), version 4.0.4.

## Results

**FIGURE 7:**
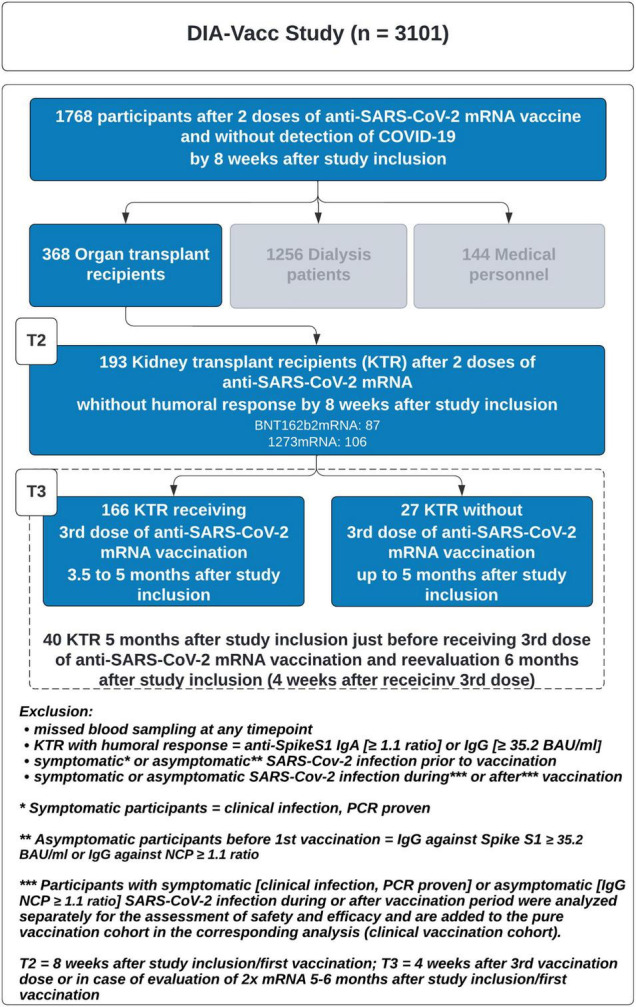
Study flow chart.

Follow-up data were available in 193 KTR (58 ± 13.6 years, 66% men, Tables 5, 6) not responding with seroconversion as defined by an insufficient humoral immune response at T2 (<1.1 ratios for anti-SpikeS1 IgA ab and <35.2 BAU/ml for IgG ab), of which 106/87 were immunized twice with 1273-mRNA or BNT162b2-mRNA, respectively. Of 193 KTR, 166 received an additional booster vaccination 3.5 to 5 months after the study started. Twenty-seven of 193 KTR did not receive any booster vaccination for up to 6 months, while 40/193 were additionally investigated as unboostered study participants around 5 months before receiving a booster vaccination and reevaluation at 6 months (Figure 7). The mean time on dialysis before transplantation is 6 years and the mean time after transplantation is 8.5 years. One in seven had been kidney transplanted before. Immunosuppressive therapy included a calcineurin inhibitor in 93%, MMF/MPA in 88%, and corticosteroids in only 47% of cases, whereas mTOR inhibitors were used in 11% and Belatacept in only 7%. Further baseline characteristics can be found in Table 5 and a schedule in Figure 1.

**TABLE 5:** Baseline characteristics of SARS-CoV-2 unexposed entire cohort corresponding to T2 non-seroconverted kidney transplant recipients and the boostered cohort corresponding to three vaccine dose kidney transplant recipients.

Variable	Category	Entire cohort	Boostered cohort
Number	Evaluable	193	166
Age (years)	Mean ± SD	58 ± 13.6	58.4 ± 13.5
Male sex	n/%	128/66.3	117/70.5
BMI (kg/m^2^)	mean ± SD	25.9 ± 5	26 ± 4.9
Cause of end stage renal disease	n/%	116/60.1	99/59.6
Diabetes-Hypertension-Vascular disease	n/%	35/18.1	32/19.3
Glomerulonephritis-Interstitial nephritis	n/%	49/25.4	41/24.7
Vasculitis	n/%	7/3.6	6/3.6
Polycystic kidney disease	n/%	25/13	20/12
Unknown	n/%	77/39.9	67/40.4
Drug treated comorbidities	n/%	176/91.2	149/89.8
Diabetes mellitus	n/%	35/18.1	32/19.3
Cardiovascular disease	n/%	170/88.1	143/86.1
Lung disease	n/%	15/7.8	11/6.6
Liver cirrhosis	n/%	3/1.6	3/1.8
Cancer	n/%	5/2.6	3/1.8
None	n/%	17/8.8	17/10.2
Time on dialysis (years)	Mean ± SD	6.1 ± 6.2	6.3 ± 6.3
Time on transplantation (years)	Mean ± SD	8.5 ± 6.3	8.2 ± 6.1
Previous transplantation	n/%	28/14.5	23/13.9
Hepatitis B vaccination failure	n/%	10/5.2	9/5.4
Flu vaccination winter 2020/2021	n/%	106/54.9	90/54.2
On immunosuppressive therapy	n/%	193/100	166/100
Corticosteroids	n/%	91/47.2	78/47
Calcineurin-inhibitor	n/%	179/92.7	154/92.8
MMF/MPA	n/%	170/88.1	146/88
mTOR-Inhibitor	n/%	22/11.4	18/10.8
Belatacept	n/%	14/7.3	12/7.2
T-cell depleting ab	n/%	0/0	0/0
B-cell depleting ab n	n/%	1/0.5	1/0.6
Other	n/%	2/1	2/1.2
**Type of vaccine**			
BNT162b2-mRNA	n/%	87/45.1	83/50
1273-mRNA	n/%	106/54.9	83/50

*The entire cohort consists of non-responding KTR after two mRNA vaccinations. Boostered cohort consists of non-responding KTR after two mRNA vaccinations who received the third vaccination.*

For this evaluation, all patients with asymptomatic* or documented symptomatic** COVID-19 disease before and during vaccination up to T3 (6 months) were excluded.

*Hepatitis B vaccination failure definition–patients with unsuccessful vaccination after at least four attempts; MMF-MPA, mycophenolate mofetil or mycophenolic acid.*

*Asymptomatic COVID-19 disease definition–neither knowledge nor symptoms of COVID-19 disease, but IgG-antibody reaction to nucleocapsid (T0, T1, T2, or T3) or the Spike protein subunit S1 (only T0) of the SARS-CoV-2 virus is positive.

**Symptomatic COVID-19 disease definition–SARS-CoV-2 PCR positive patients with clinical symptoms.

**TABLE 6:** Baseline characteristics of different vaccine combinations.

Variable		3x BNT162b2	2x BNT162b2, 1x 1273	2x 1273, 1x BNT162b2	3x 1273	2x mRNA, 1x vector	2x mRNA
Number	Evaluable	57	22	16	63	8	67
Age (years)	Mean ± SD	55.6 ± 15.5	60 ± 13.4	58.9 ± 13.3	59.9 ± 12.3	60.8 ± 7.4	57.1 ± 12.9
Male Sex	n/%	39/68.4	19/86.4	9/56.2	45/71.4	5/62.5	40/59.7
BMI (kg/m2)	Mean ± SD	25.6 ± 4.9	26.1 ± 3.7	26.3 ± 4.3	25.8 ± 4.6	28.8 ± 9.9	26.2 ± 5
Cause of end stage renal disease	n/%	36/63.2	16/72.7	12/75	29/46	6/75	44/65.7
Diabetes-hypertension-vascular disease	n/%	13/22.8	2/9.1	4/25	11/17.5	2/25	10/14.9
Glomerulonephritis-interstitial nephritis	n/%	13/22.8	9/40.9	7/43.8	9/14.3	3/37.5	21/31.3
Vasculitis	n/%	3/5.3	1/4.5	1/6.2	0/0	1/12.5	2/3
Polycystic kidney disease	n/%	7/12.3	4/18.2	0/0	9/14.3	0/0	11/16.4
Unknown	n/%	21/36.8	6/27.3	4/25	34/54	2/25	23/34.3
Drug treated comorbidities	n/%	51/89.5	21/95.5	14/87.5	56/88.9	7/87.5	60/89.6
Diabetes mellitus	n/%	14/24.6	3/13.6	2/12.5	10/15.9	3/37.5	7/10.4
Cardiovascular disease	n/%	46/80.7	21/95.5	14/87.5	56/88.9	6/75	60/89.6
Lung disease	n/%	5/8.8	0/0	1/6.2	4/6.3	1/12.5	6/9
Liver cirrhosis	n/%	2/3.5	1/4.5	0/0	0/0	0/0	0/0
Cancer	n/%	2/3.5	1/4.5	0/0	0/0	0/0	2/3
None	n/%	6/10.5	1/4.5	2/12.5	7/11.1	1/12.5	7/10.4
Time on dialysis (years)	Mean ± SD	6.3 ± 6.9	5.8 ± 3.5	8.9 ± 9.2	5.3 ± 4.5	7.7 ± 4.2	6 ± 6.8
Time on transplantation (years)	Mean ± SD	6.9 ± 6	8.4 ± 5.7	8.8 ± 7.6	9.3 ± 5.8	6.4 ± 7.3	10 ± 6.9
Previous transplantation	n/%	9/15.8	5/22.7	3/18.8	5/7.9	1/12.5	8/11.9
Hepatitis B vaccination failure	n/%	4/7	1/4.5	0/0	3/4.8	1/12.5	5/7.5
Flu vaccination winter 2020/2021	n/%	26/45.6	16/72.7	10/62.5	33/52.4	5/62.5	46/68.7
On immunosuppressive therapy	n/%	57/100	22/100	16/100	63/100	8/100	67/100
Corticosteroids	n/%	28/49.1	10/45.5	13/81.2	24/38.1	3/37.5	35/52.2
Calcineurin-inhibitor	n/%	51/89.5	19/86.4	16/100	60/95.2	8/100	63/94
MMF/MPA	n/%	49/86	19/86.4	14/87.5	57/90.5	7/87.5	62/92.5
mTOR-inhibitor	n/%	9/15.8	5/22.7	1/6.2	3/4.8	0/0	5/7.5
Belatacept	n/%	6/10.5	1/4.5	0/0	5/7.9	0/0	4/6
T-cell depleting ab	n/%	0/0	0/0	0/0	0/0	0/0	0/0
B-cell depleting ab n	n/%	0/0	0/0	0/0	1/1.6	0/0	0/0
Other	n/%	0/0	0/0	1/6.2	1/1.6	0/0	1/1.5

*BNT162b2 = BNT162b2-mRNA or tozinameran or brand name Comirnaty; 1273 = 1273-mRNA or brand name Spikevax; vector = vector vaccine consisting of AZD1222 in 5 cases and Ad26.COV2.S in 3 cases.*

For this evaluation, all patients with asymptomatic* or documented symptomatic** COVID-19 disease before and during vaccination up to T3 (6 months) were excluded.

*Hepatitis B vaccination failure definition–patients with unsuccessful vaccination after at least four attempts; MMF-MPA = mycophenolate mofetil or mycophenolic acid.*

*Asymptomatic COVID-19 disease definition–neither knowledge nor symptoms of COVID-19 disease, but IgG-antibody reaction to nucleocapsid (T0, T1, T2, or T3) or the Spike protein subunit S1 (only T0) of the SARS-CoV-2 virus is positive.

**Symptomatic COVID-19 disease definition–SARS-CoV-2 PCR positive patients with clinical symptoms.

### Study End Points

#### Humoral and Cellular Response Rates 6 Months After the First Vaccination (T3) in Kidney Transplant Recipients Not Responding Previously With Seroconversion 8 Weeks After the First Vaccination

Seroconversion results of initially non-responsive KTR based on different third vaccine types (no vaccine vs. BNT162b2-mRNA vs. 1273-mRNA vs. vector vaccine) varied between 13 and 50% (Table 1).

Four weeks after a third vaccination, humoral response in terms of seroconversion of anti-SpikeS1 IgG or IgA was observed in 29.8 and 36.4% of KTR vaccinated three times with BNT162b2-mRNA or twice with BNT162b2-mRNA plus once using 1273-mRNA. In contrast, the seroconversion rate of those vaccinated with 1273-mRNA was 50 and 49.2%, depending on the vaccine combination used (2x 1273-mRNA plus 1x BNT162b2-mRNA and 3x 1273-mRNA, respectively). Comparing all 3-fold vaccine combinations, however, statistical significance was missed, but a strong trend emerged (*p* = 0.092, Table 1). Regarding the seroconversion of anti-SpikeS1 IgG, response rates of 21.1 (3x BNT162b2-mRNA) and 27.3% (2x BNT162b2-mRNA plus 1x 1273-mRNA) were observed. In contrast, again higher seroconversion rates of 43.8 (2x 1273-mRNA plus 1x BNT162b2-mRNA) and 46% (3x 1273-mRNA) were achieved with the 1273-mRNA-based vaccine combinations. The difference comparing all 3-fold vaccine combinations was statistically significant (*p* = 0.025, Table 1). Looking at anti-RBD IgG when comparing all 3-fold vaccine combinations performed, there was no significant difference (Table 1). BNT162b2-mRNA based combinations showed rates of 19.3 (3x BNT162b2-mRNA) and 22.7% (2x BNT162b2-mRNA plus 1x 1273-mRNA) and 1273-mRNA based combinations of 18.8 and 31.7% (2x 1273-mRNA plus 1x BNT162b2-mRNA and 3x 1273-mRNA, respectively). Considering anti-SpikeS1 IgA conversion rates, again there was no significant difference in response rates, considering all 3-fold vaccine combinations (*p* = 0.524, Table 1) of 21.1 (3x BNT162b2-mRNA), 27.3 (2x BNT162b2-mRNA plus 1x 1273-mRNA), 25 (2x 1273-mRNA plus 1x BNT162b2-mRNA) and 33.3% (3x 1273-mRNA). The T-cellular immune response could only be examined in part in the KTR and borderline non-significant differences were found (*p* = 0.06, again in regard to a comparison of all 3-fold vaccine combinations). Trend differences, analogous to humoral seroconversion rates, could thereby be confirmed in distinct vaccine combinations. A tendency toward a better response rate for triple vaccinations with 1273-mRNA is 37.5%, compared to 6.2% for triple vaccinations with BNT162b2-mRNA (Table 1). The number of measured KTR who were vaccinated heterologically using 2x mRNA plus 1x vector vaccine is small. Response rates resemble spontaneous delayed seroconversion rates of the unboostered KTR (Table 1).

The unboostered KTR showed combined delayed seroconversion rates of anti-SpikeS1 IgA or IgG of 16.4%, 6 months after the first mRNA vaccination. Hereby, the rates for anti-SpikeS1 IgG and anti-RBD IgG alone were 14.9 and 9%, respectively. Humoral immunity as anti-SpikeS1 IgA was 10.4% in this group and T-cellular immune reaction measured by interferon-γ release assays showed a spontaneous delayed response rate of 20% (Table 1).

Next, we explored whether IgG seroconversion success after booster vaccination depends on the initial or booster vaccine type, immunosuppressive agents, comorbidities, etc. as evaluated before for seroconversion success after two vaccinations (6). Those were further examined *via* a logistic regression approach.

#### Predictors for Seroconversion After a Third mRNA Vaccination in Kidney Transplant Recipients, Not Responding Previously With Seroconversion 8 Weeks After the First Vaccination

Using logistic regression analysis, we found that a humoral response after revaccination was 23% more likely if the primary vaccine was 2x 1273-mRNA than 2x BNT162b2-mRNA (OR = 4.420, 95% CI [1.208–16.173], *p* = 0.025, Table 3 and Figure 2). The median antibody titers when 1273-mRNA was used as the exclusive vaccine type were 28.1 BAU/ml for anti-SpikeS1 IgG compared with the titers of 3.8 BAU/ml when BNT162b2-mRNA was used (Table 2). The effect of the third vaccine type was non-significant [$\chi_{\left(2\right)}^{2}$ = 0.41, *p* = 0.639, Table 4]. Neither differed time between the second and third vaccination, gender, age, time after transplantation, hepatitis B vaccination failure on dialysis, and diabetes mellitus as comorbidity significantly in the multiple logistic regression analysis. Seroconversion success was observed in 53 vs. 20% of patients with a weak (3.2–35.2 BAU/ml) vs. negative (<3.2 BAU/ml) anti-SpikeS1 IgG response at T2, respectively (OR 1.360, 95% CI [1.167–1.584], *p* = 0.001, Table 4). The anti-SpikeS1 IgG threshold distributions were similar for primary vaccine subgroups [$\chi_{\left(1\right)}^{2}$ = 0.23, *p* = 0.630], but differed significantly after revaccination [$\chi_{\left(2\right)}^{2}$ = 10.76, *p* = 0.005]. KTR with primary 1273-mRNA were consistently more likely to respond with seroconversion than those with primary BNT162b2-mRNA: 66.7 vs. 41.2% (*p* = 0.074) in the weak anti-SpikeS1 IgG group and 32.1 vs. 6.3% success rate (*p* = 0.003) in the negative anti-SpikeS1 IgG group. This overall advantage for primary 1273-mRNA compared to BNT162b2-mRNA vaccination is also appreciated in Figure 5C, where the orange line (1273-mRNA) is above the green line (BNT162b2-mRNA) for most patients’ values except the very few in the highest range of the weak anti-SpikeS1 IgG group. Looking at different mRNA-based vaccine combinations, all vaccine combinations show a booster success correlation to a weak compared to no anti-SpikeS1 IgG reaction after two vaccinations, since all lines showed an incline (Figure 5D). As indicated by Figure 5D, those ostensibly relying on 3x 1273-mRNA (orange line) or heterologous 2x 1273-1xBNT162b2-mRNA (blue line) vaccination lead not only to higher seroconversion rates but also to markedly higher IgG levels independent on no or a weak response after two vaccinations.

A similar picture indicating advantages for 1273-mRNA emerges when looking at anti-RBD IgG seroconversion rates considering the primary vaccine types (Figure 6C–1273-mRNA in orange and BNT162b2-mRNA in green). With regard to RBD-level changes after different vaccine combinations, 3xBNT162b2-mRNA appears to be the least effective and without correlation to some weak RBD-level induction after two vaccinations (Figure 6D–green line without incline). RBD-stimulation after three vaccinations appeared positively related to all other vaccine combinations (Figure 6D–orange, black, and blue inclining lines). For RBD-level changes, the most effective vaccine combination appears to be the heterologous 2x 1273-1xBNT162b2-mRNA (blue line), while 3x 1273-mRNA and heterologous 2xBNT162b2-/1x1273-mRNA were intermediate (orange and black lines in Figure 6D).

Logistic regression revealed that MMF/MPA in the context of immunosuppressive therapy was borderline significant as another predictor of seroconversion after a third mRNA vaccination in KTR. Thereby, the use of MPA/MMF is associated with a worse seroconversion rate (OR = 7.086, 95% CI [0.917–54.730], *p* = 0.060, Table 4).

Anti-Spike S1 IgG seroconversion rates as a function of the vaccine type used for the first and second vaccination, 4 weeks after the latter (T2, all without positive seroconversion) and then again 4 weeks after the third vaccination (T3) are depicted in Figure 2. We demonstrate higher response rates (blue lines vs. non-responders in yellow lines) in KTR primarily immunized with 1273-mRNA compared to BNT162b2-mRNA.

#### Analysis of Humoral and T-Cellular Titer Levels 6 Months After the First Vaccination (T3) in Kidney Transplant Recipients Not Responding Previously With Seroconversion 8 Weeks After the First Vaccination

Titer levels, two (T2) and six (T3) months after initial vaccination, concerning the humoral and T-cellular vaccination response are shown with median and IQR, for all vaccine combinations investigated, in Table 2.

Comparing all triple mRNA vaccine combinations, median anti-SpikeS1 IgG levels between T3 and T2 evaluations differed significantly. In BNT162b2-mRNA based vaccination regimes, there was an increase from 3.2 to 3.8 (3x BNT162b2-mRNA) or from 3.2 to 3.5 BAU/ml (2x BNT162b2-mRNA plus 1x 1273-mRNA). In 1273-mRNA-based vaccination regimes, IgG titers increased from 3.2 to 28.1 (3x 1273-mRNA) and from 3.2 to 22.8 BAU/ml (2x 1273-mRNA plus 1x BNT162b2-mRNA). Heterologous combination with vector vaccine showed no median increase in titer. Interestingly, after 2x mRNA vaccination, unboostered KTR revealed some delayed (the latter because without booster or indication of SARS-CoV-2 exposure) increase from 3.2 to 3.6 BAU/ml.

Regarding anti-RBD IgG (indicating neutralizing activity against the SARS-CoV-2 virus variants alpha to delta), statistically significant median titer increases were only seen in the 3-fold 1273-mRNA vaccinated KTR group (3.9–7.5% inhibition, *p* < 0.001), while all other triple mRNA or vector vaccinated or unboostered KTR did not show a significant increase in median RBD titers.

Triple mRNA vaccinated KTR demonstrated increasing median titers from T2 to T3 with respect to anti-SpikeS1 IgA. The greatest increase in titer was seen again in the 3-fold 1273-mRNA vaccinated KTR, from 0.3 (IQR.2–0.5) to 0.5 ratio (IQR.3-1.4, *p* < 0.001). The group of heterologous vector vaccinated KTR showed no increase in median titers similar to those vaccinated only two times.

T-cellular immunity results were not available for all triple-vaccinated KTR at both time points. Interestingly, in 3x BNT162b2-mRNA vaccinated KTR, median titer decreased from 13.3 to 8.3 mIU/ml but showed an increase in the range of values (IQR from 0.9–38.9 to 1.3–60.3). In contrast, after 2x BNT162b2-mRNA plus 1x 1273-mRNA, there was a median titer increase from 0 to 17.2 mIU/ml. No IGRA measurements were available for 3x 1273-mRNA plus 1x BNT162b2-mRNA vaccinated KTR at T3. The only group in which the 75% percentile of levels (upper limit of the IQR) was above the positive test threshold, and thus another 25% of the values above it, were 3x 1273-mRNA vaccinated KTR (titer increased from 23.6 to 28.6 mIU/ml while IQR spread from 3.7–151.4 to 6.1–200.4). Even though heterologous vector vaccinated KTR formally show an increase in median titers (7.1 to 11.9 mIU/ml), the IQR on the other hand reduced from 0–183.6 to 7.1–31.3 at the respective time points. In unboostered KTR with 2x mRNA vaccination, both the median titers decreased from 26.1 to 23.7 mIU/ml and the IQR decreased from 11.8–444.3 to 5.8–107.

For better illustration, we categorized the anti-SpikeS1 IgG (Table 3 and Figure 3A) and anti-RBD IgG titer levels (Table 3 and Figure 4A) into six intervals each and plotted them accordingly at times T2 and T3. The exact limits of the intervals (referred to as levels) can be found in the corresponding tables mentioned above. Regarding anti-SpikeS1 IgG levels, Figure 3A shows the changed distribution of the different IgG titer levels 4 weeks after the third vaccination at T3 (brown, indicating positive seroconversion of 34.2%) compared to before at T2 (green) in primary non-responders. In addition to this, we counted numbers of positive level changes graduating the extent of a positive response. To achieve a seroconversion from an initial negative test result, a maximum increase of 2 level changes is needed (minimally, if previously in the gray area, only 1 level change in increase is needed). These level changes are displayed in Figure 3B for anti-SpikeS1 IgG, subdivided into the primary vaccines in Figure 3C and subdivided into the different 3-fold mRNA vaccine combinations in Figure 3D of the corresponding plot. The level changes of anti-SpikeS1 IgG measurements of ≥2 add up to 34.2% (Figure 3B). When analyzed by the corresponding primary vaccination regimen, there is a proportional advantage in the use of 1273-mRNA as the initial vaccine for KTR (Figure 3C), as there was for the overall response for each level change. This is further emphasized in the graphical representation of the level changes subdivided into the different 3-fold mRNA vaccine combinations (Figure 3D). Hereby, the individual level changes show broader corresponding bands for combinations containing 1273-mRNA, especially those with 1273-mRNA as the primary (2x) vaccine regimen.

The serological response of anti-RBD IgG at T3 compared to T2 (level ≥ 2) indicating neutralizing capacity is less than one quarter (24.8%, Figure 4A) and corresponds exactly to the level changes (Figure 4B). Taking into account the level changes subdivided into the primary vaccine types, it is striking that 31.2% of the primary 1273-mRNA vaccinated KTR make a level change of ≥2 levels, whereas this is only the case in 12.4% of the corresponding primary BNT162b2-mRNA vaccinated KTR (*p* = 0.08). Again, the subdivision according to 3-fold mRNA vaccine combinations shows that the bands of the higher-level changes are broader in the vaccine combinations containing 1273-mRNA, especially those with 1273-mRNA as the primary (2x) vaccine regimen.

## Discussion

Our study in 193 non-responding KTR after 2x mRNA vaccination showed that successful seroconversion after the third vaccination is dependent on the choice of vaccine type, the level of weak (below positivity threshold) IgG titer stimulation after 2x mRNA vaccination, and the use/lack of MMF/MPA as an immunosuppressive drug.

The dependence of seroconversion success on the vaccine type was particularly evident when comparing the homologous vaccine combinations of 3-fold 1273-mRNA and 3-fold BNT162b2-mRNA (Table 1). This result in non-responding KTR is predominantly driven *via* increased rates of antiSpikeS1 IgG antibody induction. It is consistent with former studies by us and others examining organ transplant recipients after two mRNA vaccinations (6, 7, 9), in which markedly higher seroconversion rates had been shown in 1273-mRNA vaccinated kidney transplant recipients (6) (49 vs. 26% in BNT162b2-mRNA). In our current study, we unexpectedly found that 1273-mRNA compared to BNT162b2-mRNA, as the third vaccine, is not critical for this improved seroconversion rate but rather the original use of 1273-mRNA as the primary vaccine type for the first two vaccinations. When BNT162b2-mRNA is being used as the third vaccine after primary 1273-mRNA immunizations, equivalent IgG seroconversion rates up to almost 50% compared to 3x 1273-mRNA can be achieved. Independent of the choice of the third mRNA vaccine type, seroconversion rates with BNT162b2-mRNA as the primary vaccine regimen did not even reach seroconversion rates of 25%. Apart from seroconversion rates, these results showing dependence on the primary vaccine type were also mimicked considering the extent of antiSpikeS1 IgG titer level changes as quantified by intervals in our study. While high RBD-IgG level changes after the third vaccination seemed to be more frequent when 1273-mRNA compared to BNT162b2-mRNA has been used as the primary vaccine type supporting these data, RBD-IgG or IgA antibody seroconversion rates were not similarly influenced by mRNA vaccine type. T-cell immune response measurements support the success of 3x 1273-mRNA vaccinations but were not frequently done enough to compare the different vaccine combinations statistically.

The simplest explanation for the higher immunogenicity of the 1273-mRNA vaccine in KTR was and still is the 3-fold higher mRNA dose, better thermostability, and easier handling of the 1273-mRNA compared to the BNT162b2-mRNA vaccine. Nevertheless, differences in antigenic motifs or mRNA modifications and different formulations may also play a role. It remains unclear, why, this effect of 1273-mRNA is especially important in the primary immunization process and can be less attributed to the third vaccine dose in our study. A prolonged vaccination interval as a potential differential influence on seroconversion has already been demonstrated for the BNT162b2-mRNA vaccine in the United Kingdom when the interval was extended from 3–4 to 6–14 weeks (20, 21) also had to be excluded. In contrast to the United Kingdom study, our study did not show any difference in the levels of anti-SpikeS1 IgG vaccination response between early and late third vaccination time points (period 8–12 weeks after the second vaccination, see horizontal lines in Figures 5A, 6A than lines ascending with increasing time interval [over the x-axis to the right]). The afore-mentioned concept of T-cell exhaustion (21) cannot be replicated in the present data set, but the time intervals chosen in our study were also less heterogeneous than in the United Kingdom study. For interpretation of our and the United Kingdom data, our finding of a spontaneous seroconversion rate in up to 1/6 of the KTR, immunized with only two vaccine doses, also suggests a severely delayed immunological responsiveness under immunosuppressive therapy. The small group of KTR immunized with heterologous 2x mRNA and 1x vector vaccine showed similar immune response rates in any test system examined as unboostered KTR with only 2x mRNA. While the interpretation of these data needs to be done with caution, this limited effect as a boostering vaccine has already been described in the literature for vector vaccines and is confirmed in the present population (22).

In line with previous studies for other humoral test systems (11), weak (below the positivity level of the test but above the detectability threshold), compared to negative responders were more likely to show seroconversion after the third vaccination. Taking into account all limitations of the test systems with gray range, detection thresholds, and linearity of the measurement ranges, we believe that anti-SpikeS1 IgG titers >3.2 and <35.2 BAU/ml define a sub-cohort within the immunosuppressed KTR that can be distinguished from an immunologically almost anergic group of non-responders (≤3.2 BAU/ml). Consistent with low humoral response rates 4 weeks after two mRNA vaccine doses, there is no evidence of increased rejection rates in COVID-19-diseased (23) or vaccinated (24) kidney transplant recipients. However, the present results show that serologic testing of the vaccination response in immunocompromised KTR makes sense defining cohorts with either successful seroconversion as well as non-responding patients with better or worse chances for a successful booster vaccination. In this sub-cohort of non-responding KTR with better chances for successful booster vaccination, no potentially risky reductions in immunosuppressive therapy, as already proposed by others (25), may be necessary to achieve high seroconversion rates as shown here. Hereby it also can be considered that successful seroconversion is accompanied by, albeit relative (depending on the variants of the virus), protection from severe or fatal infections.

Others and we demonstrated that besides the vaccine type used MMF/MPA, as part of the standard immunosuppressive therapy in KTR, predicts humoral response to mRNA COVID-19 vaccines (6, 26, 27). While this has been described as a predictor of humoral response after the second vaccine dose, we interpret MMF/MPA intake, despite borderline significance, as a predictor of humoral response also to a third vaccine dose. An unfavorable dose-dependent effect of MMF/MPA has been suggested (27). MMF/MPA as an anti-metabolite impairs not only B-cell proliferation and maturation into plasma blasts (28) but also expansion and activation of B cells (29, 30). Mechanistically, inhibition of the STAT3 pathway in particular is thought to be responsible for impaired differentiation of B-cells up to immunoglobulin secretion in the bone marrow (31, 32). The impact of MMF/MPA on B cells in an antigen-specific context up to the impairment of spike-specific CD27++CD38+plasma blast formation was recently shown by colleagues for the first time in a clinical setting (25). They also showed, that not only MMF/MPA dose modification could lead to an improved immune response but a temporary hold of MMF/MPA for 5 weeks is a feasible option to facilitate immunogenicity KTR (25). Although no increased rejection rates have been reported, this certainly remains the biggest concern of the approach proposed and should only be considered in the almost anergic non-responding group after 2x vaccinations (≤3.2 BAU/ml).

Limitations of our approach include the non-randomized observational nature of our study and potential bias in patient selection, who participated only if interested in SARS-CoV-2 vaccination. For KTR with similar characteristics, these results should still be applicable but could also be confirmed in prospective controlled randomized trials. Another limitation is the lack of a detailed characterization of the T-cell mediated immune response as well as functional virus-related neutralization tests. Nevertheless, these tests are extremely work-intensive and not at all part of a standard diagnostic procedure for immune monitoring after vaccination and are not suited (especially not in an observational diagnostic study) to prove a causal link between vaccination-related immune response and disease incidence/mortality or even vaccination efficacy. Hereby it needs to be considered that even functional neutralization tests being performed *in vitro* never reflect real-life conditions, where in addition to the current immune status of the host, the route of viral transmission (inhalation vs. nasal mucosal contact), the viral load, virulence factors of the pathogen, and the type of viral variants (wild type, alpha, delta, omicron, etc.) play a role in the incidence and time course of the disease. With all these limitations in mind, Dolscheid-Pommerich and colleagues already described some correlations between the quantitative anti-SARS-CoV-2 IgG ELISA (as is also used here) and virus neutralization activity *in vitro* (33). This correlation may be true for our corresponding study period, while it is not applicable for the later appearing Omicron SARS-CoV-2 variant (VOC strain B.1.1.529).

In conclusion, this study provides important evidence for the use of 1273-mRNA as the primary mRNA vaccine type for immunocompromised KTR, which not only positively influence seroconversion after 2x vaccination but also improves the chance of seroconversion in non-responding KTR independent of the choice of the third mRNA vaccine. In addition, serologic testing should be performed subsequently in this vulnerable patient population to monitor and partly predict vaccination response. Weak (below positivity level) responders after two mRNA vaccinations have a good seroconversion chance after additional booster vaccinations despite current immunosuppressive therapy. Heterologous mRNA vaccine use as a third vaccination in non-responding KTR may be especially useful when BNT162b2-mRNA was used as the primary immunization. A temporary MMF/MPA withdrawal could be taken into consideration especially in KTR with no IgG response (≤3.2 BAU/ml) after two mRNA vaccinations.

## Data Availability Statement

The raw data supporting the conclusions of this article will be made available by the authors, without undue reservation.

## Ethics Statement

The studies involving human participants were reviewed and approved by the Ethical Institutional Review Boards at Technische Universität Dresden (TU Dresden) responsible for the coordinating investigator (BO-EK-45012021), as well as at the University of Leipzig (046/21-lk) and Saxon Medical Association (Sächsische Landesärztekammer—EK-BR-10/21-1) responsible for further participating trial sites. The patients/participants provided their written informed consent to participate in this study.

## Author Contributions

JSt and CH contributed to the study design, data collection and interpretation, and drafting of the manuscript. JSc, CK, HS, RM, AK, and TT were involved in data acquisition and collection and study organization or contributed to data interpretation. AK and RM were involved in the statistical analysis or data management of the study. JSt, JSc, CK, HS, and CH were involved in patient recruitment and data collection. All authors have approved the final version for submission.

## Conflict of Interest

The authors declare that the research was conducted in the absence of any commercial or financial relationships that could be construed as a potential conflict of interest.

## Publisher’s Note

All claims expressed in this article are solely those of the authors and do not necessarily represent those of their affiliated organizations, or those of the publisher, the editors and the reviewers. Any product that may be evaluated in this article, or claim that may be made by its manufacturer, is not guaranteed or endorsed by the publisher.

## Further Supporters

*D. Michael Albrecht, Medizinischer Vorstand (Sprecher), Universitätsklinikum Carl Gustav Carus Dresden*

*Thomas Grünewald, Chemnitz, Vorsitzender Sächsische Impfkommission*

*Patricia Klein, Ärztliche Geschäftsführerin, Sächsische Landesärztekammer Körperschaft des öffentlichen Rechts*

*Marcus Strotkötter, Senior Operations Officer for Vaccination Center and Mobile Vaccination Teams at DRK Landesverband Sachsen e.V.*

*EUROIMMUN Medizinische Labordiagnostika AG, Lübeck, Germany provided antibody ELISAs and interferon-gamma release assays for this study.*
